# Association between anemia and grip strength indices combined with anthropometry in the Korean population

**DOI:** 10.1038/s41598-023-45985-5

**Published:** 2023-10-28

**Authors:** Bum Ju Lee, Jeong Hee Chi

**Affiliations:** 1 Digital Health Research Division, Korea Institute of Oriental Medicine, 1672 Yuseong‑daero, Yuseong‑gu, Daejeon, 34054 Korea; 2 Department of Computer Science and Engineering, Konkuk University, Seoul, Korea

**Keywords:** Epidemiology, Public health

## Abstract

Anemia is common in older people and is associated with low hand grip strength (HGS). However, there is no study of the association of anemia with both absolute and various relative HGS indices. Therefore, the objectives of this study are to examine the association of anemia with absolute and relative HGS indices and to evaluate whether the relative HGS indices are useful as risk indices of anemia. In this large-scale cross-sectional study, we analyzed the association of anemia with anthropometric indices, absolute HGS indices, and relative HGS indices using both crude and adjusted binary logistic regression models. A total of 24,022 participants were included in the final analysis. Anemia was defined as a blood hemoglobin concentration of less than 13.0 g/dL for men, less than 11.0 g/dL for pregnant women, and less than 12.0 g/dL for nonpregnant women. We considered covariates such as residential area, marital status, education level, occupation, household income, alcohol consumption, smoking status, muscular exercise, walking exercise, blood pressure, and age for men, while for women, we also included menopause. The mean ages of the subjects in this study were 51.89 ± 0.19 years in the nonanemic group and 66.56 ± 0.61 in the anemic group in men and 52.55 ± 0.19 in the nonanemic group and 51.85 ± 0.44 in the anemic group in women. The number of anemic patients was 570 (5.5%) in men and 1638 (12%) in women. The mean HGS value in the dominant hand was 38.97 ± 0.11 in the nonanemic group and 30.84 ± 0.38 in the anemic group in men and 22.64 ± 0.07 in the nonanemic group and 21.85 ± 0.16 in the anemic group in women. Age was highly associated with anemia in men but not in women. Absolute HGS indices, such as the dominant HGS index and both HGS indices, were negatively associated with anemia in both sexes in all crude and adjusted models. All relative HGS indices were associated with anemia in men, but in women, only dominant HGS divided by height and both HGS divided by height were related to anemia status in all models. The associations between anemia and absolute and relative HGS indices were significantly high in men, while in women, these associations were of moderate strength. Anemia was negatively associated with HGS in the Korean population. The strengths of the associations of anemia with the absolute and relative HGS indices showing the highest association with anemia were similar. Therefore, there is no need to use relative HGS indices as risk factors for anemia, and absolute HGS indices, as easily obtained and cost-effective measurement, are suitable.

## Introduction

Anemia is a major public disease worldwide and contributes to disability^1–4^, mortality^1,2,5,6^, and low quality of life^2,4,6^, notably in young children, adolescent girls, and older people^5,7^. Anemia affects approximately 1/3 of the world’s population^5,8^ and is the consequence of various risk factors influenced by sociodemographic, biological, and ecological characteristics^7^. Among demographic and socioeconomic characteristics, a variety of risk factors and causes of anemia have been identified, such as age, pregnancy, income, education level, micronutrient deficiency, chronic and inherited diseases, and obesity^7,9,10^. Biologically, anemia is caused by an imbalance in the loss and production of red blood cells or deficient erythropoiesis due to inherited red blood cell disorders, lack of nutrition, inflammation, blood loss, and hemolysis^5^.

Recently, several studies have suggested that anemia or low hemoglobin levels are closely associated with hand grip strength (HGS)^1,3,4,11–18^. For example, Hirani et al.^1^ revealed that every 1 g/dL increase in hemoglobin significantly decreased the risk of HGS, and HGS had a strongly positive association with hemoglobin levels in both cross-sectional and longitudinal analyses. Generally, HGS is influenced by hypertension and diabetes^19^, anthropometry or obesity^9^, aging^3,9^, HDL-C^20^, forced expiratory volume^20^, malnutrition^21^, fatigue^4,15,18^, dyspnea^15,18^, muscular exercise^1^, inflammation^4,11,22,23^, and anemia^1,3–18,24^. Anemia affects cognitive performance and physical performance, such as HGS and walking speed^1,24^. Conversely, anemia treatments may improve HGS, walking speed, and cognitive status^24^. Although the mechanism linking anemia and low HGS is unclear, however, due to the complexity of associated biological and pathological factors^4,15^, subjects with low hemoglobin levels generally have lower muscle and HGS strength than those with normal hemoglobin levels, and anemia is associated with inflammation, which has been identified as a crucial etiology of the disease^1,4,11,22^. Therefore, examination of absolute and relative HGS indices may have clinical significance in terms of their associations with anemia and inflammation.

Until now, although many studies have reported that HGS indices are related to anemia or hemoglobin levels^1,3–18,24^, these studies examined only the association between absolute HGS indices and anemia. There is no study of the association of anemia with both absolute and various relative HGS indices. Therefore, the objectives of this study are to examine the association of anemia with both absolute HGS indices and ten relative HGS indices combined with anthropometry and to evaluate whether the relative HGS indices are useful as risk indices of anemia. This is the first study to report the association of anemia with absolute and relative HGS indices; the study found that although relative HGS indices were associated with anemia, the indices were not necessary for assessing the risk of anemia due to the accessibility of other cost-effective methodologies.

## Results

### Demographic characteristics of the subjects included in this study

The mean ages of the subjects enrolled in this study were 51.89 ± 0.19 in the nonanemic group and 66.56 ± 0.61 in the anemic group in men and 52.55 ± 0.19 in the nonanemic group and 51.85 ± 0.44 in the anemic group in women (Table 1). The number of anemic patients was 570 (5.5%) in men and 1638 (12%) in women. The mean HGS value in the dominant hand was 38.97 ± 0.11 in the nonanemic group and 30.84 ± 0.38 in the anemic group in men and 22.64 ± 0.07 in the nonanemic group and 21.85 ± 0.16 in the anemic group in women. Age was highly associated with anemia in men (p < 0.001) but not in women (p = 0.103). In men, anemia was highly related to education level, occupation, household income, smoking, muscular exercise, diastolic blood pressure (DBP), hemoglobin level, red blood cell (RBC) count, all anthropometric indices, and all absolute and relative HGS indices (p < 0.001). Systolic blood pressure (SBP) was also associated with anemia (p = 0.002). In women, anemia was highly associated with education level, menopause, DBP, hemoglobin level, RBC count, all anthropometric indices (except for height), absolute HGS indices, DHGS-HT, and BHGS-HT (p < 0.001). Additionally, marital status, occupation, household income, and weekly walking exercise were associated with anemia. Anemia was not associated with dominant hand or residential area in either men or women or alcohol consumption, muscular exercise, smoking, SBP, or most relative HGS indices in women.

**Table 1:** Associations of anemia with anthropometric indices and absolute and relative HGS indices among men.

Variables	Crude		Model 1		Model 2	
	OR (95% CI)	p value	Adj. OR (95% CI)	Adj. p value	Adj. OR (95% CI)	Adj. p value
Age	3.69 (3.23–4.21)	< 0.001				
Anthropometrics						
Height	0.56 (0.51–0.62)	< 0.001	0.97 (0.87–1.09)	0.591	1.05 (0.93–1.18)	0.410
Weight	0.44 (0.39–0.50)	< 0.001	0.68 (0.59–0.79)	< 0.001	0.72 (0.63–0.84)	< 0.001
Body mass index	0.58 (0.51–0.66)	< 0.001	0.69 (0.60–0.79)	< 0.001	0.70 (0.61–0.81)	< 0.001
Waist circumference	0.75 (0.67–0.84)	< 0.001	0.74 (0.66–0.83)	< 0.001	0.75 (0.67–0.85)	< 0.001
Waist-to-height ratio	0.95 (0.85–1.07)	0.422	0.75 (0.66–0.84)	< 0.001	0.74 (0.65–0.83)	< 0.001
Absolute HGS						
Dominant HGS	0.35 (0.32–0.39)	< 0.001	0.58 (0.51–0.66)	< 0.001	0.63 (0.55–0.72)	< 0.001
Both HGS	0.35 (0.32–0.39)	< 0.001	0.57 (0.50–0.65)	< 0.001	0.63 (0.55–0.72)	< 0.001
Relative HGS						
DHGS-HT	0.37 (0.34–0.41)	< 0.001	0.59 (0.53–0.67)	< 0.001	0.64 (0.56–0.72)	< 0.001
BHGS-HT	0.37 (0.33–0.41)	< 0.001	0.59 (0.52–0.66)	< 0.001	0.64 (0.56–0.72)	< 0.001
DHGS-WT	0.55 (0.50–0.62)	< 0.001	0.82 (0.73–0.92)	0.001	0.87 (0.77–0.98)	0.024
BHGS-WT	0.56 (0.50–0.62)	< 0.001	0.82 (0.73–0.92)	0.001	0.87 (0.77–0.99)	0.030
DHGS-BMI	0.47 (0.42–0.53)	< 0.001	0.80 (0.70–0.91)	0.001	0.87 (0.76–0.99)	0.041
BHGS-BMI	0.47 (0.42–0.53)	< 0.001	0.80 (0.70–0.91)	0.001	0.87 (0.76–1.00)	0.050
DHGS-WC	0.41 (0.37–0.46)	< 0.001	0.73 (0.63–0.83)	< 0.001	0.79 (0.68–0.92)	0.002
BHGS-WC	0.41 (0.37–0.46)	< 0.001	0.72 (0.63–0.83)	< 0.001	0.80 (0.69–0.92)	0.002
DHGS-WHtR	0.39 (0.35–0.44)	< 0.001	0.72 (0.62–0.83)	< 0.001	0.79 (0.68–0.92)	0.003
BHGS-WHtR	0.39 (0.35–0.44)	< 0.001	0.72 (0.62–0.83)	< 0.001	0.80 (0.69–0.93)	0.004

OR and p values were obtained from the crude and adjusted analyses using complex sample binary logistic regression. Odds ratios were estimated with 95% confidence intervals.

Model 1 was adjusted for age.

Model 2 was adjusted for residential area, marital status, education level, occupation, household income, alcohol consumption, smoking status, muscular exercise, walking exercise, blood pressure, and age.

*HGS* handgrip strength, *DHGS* dominant hand grip strength, *BHGS* both hand grip strength, *HT* height, *WT* weight, *BMI* body mass index, *WC* waist circumference, *WHtR* waist-to-height ratio, *OR* odds ratio, *CI* confidence interval.

### Association of anemia with absolute and relative HGS

In men (Table 2), all anthropometric indices were negatively associated with anemia, except for WHtR in the crude analysis and height in adjusted Models 1 and 2. Two absolute HGS indices, “Dominant HGS” and “Both HGS”, were highly associated with anemia in all models (odds ratio (OR) = 0.63 [0.55–0.72], adjusted p < 0.001 in Model 2). All relative HGS indices were related to anemia, and notably among them, DHGS-HR and BHGS-HT were highly related to anemia (OR 0.64 [0.56–0.72], adjusted p < 0.001 in Model 2). In women (Table 3), absolute HGS indices and anthropometric indices except for height were negatively associated with anemia in all models. Among the relative HGS indices, only DHGS-HR and BHGS-HT were negatively associated with the disease in all models (OR 0.87 [0.82–0.93], p < 0.001 and OR 0.86 [0.81–0.92], p < 0.001 in Model 2).

**Table 2:** Associations of anemia with anthropometric indices and absolute and relative HGS indices among women.

Variables	Crude		Model 1		Model 2	
	OR (95% CI)	p value	Adj. OR (95% CI)	Adj. p value	Adj. OR (95% CI)	Adj. p value
Age	0.95 (0.89–1.01)	0.107				
Anthropometrics						
Height	0.98 (0.92–1.04)	0.499	0.93 (0.87–1.00)	0.054	0.97 (0.90–1.05)	0.413
Weight	0.81 (0.76–0.86)	< 0.001	0.81 (0.76–0.86)	< 0.001	0.84 (0.79–0.90)	< 0.001
Body mass index	0.81 (0.76–0.86)	< 0.001	0.81 (0.76–0.86)	< 0.001	0.83 (0.78–0.89)	< 0.001
Waist circumference	0.81 (0.77–0.86)	< 0.001	0.81 (0.76–0.86)	< 0.001	0.81 (0.76–0.87)	< 0.001
Waist-to-height ratio	0.84 (0.79–0.89)	< 0.001	0.82 (0.77–0.88)	< 0.001	0.81 (0.75–0.87)	< 0.001
Absolute HGS						
Dominant HGS	0.86 (0.81–0.91)	< 0.001	0.81 (0.76–0.86)	< 0.001	0.87 (0.81–0.93)	< 0.001
Both HGS	0.85 (0.80–0.91)	< 0.001	0.80 (0.75–0.85)	< 0.001	0.86 (0.80–0.92)	< 0.001
Relative HGS						
DHGS-HT	0.85 (0.80–0.90)	< 0.001	0.81 (0.76–0.87)	< 0.001	0.87 (0.82–0.93)	< 0.001
BHGS-HT	0.84 (0.79–0.90)	< 0.001	0.80 (0.75–0.86)	< 0.001	0.86 (0.81–0.92)	< 0.001
DHGS-WT	0.97 (0.91–1.03)	0.313	0.94 (0.88–1.01)	0.082	0.98 (0.92–1.05)	0.598
BHGS-WT	0.97 (0.91–1.03)	0.274	0.94 (0.88–1.00)	0.062	0.98 (0.91–1.05)	0.501
DHGS-BMI	0.97 (0.91–1.04)	0.424	0.94 (0.87–1.00)	0.069	0.98 (0.91–1.05)	0.544
BHGS-BMI	0.97 (0.91–1.04)	0.389	0.93 (0.87–1.00)	0.052	0.97 (0.91–1.05)	0.460
DHGS-WC	0.97 (0.90–1.03)	0.318	0.92 (0.85–1.00)	0.039	0.98 (0.90–1.06)	0.548
BHGS-WC	0.96 (0.90–1.03)	0.280	0.92 (0.85–0.99)	0.027	0.97 (0.90–1.05)	0.455
DHGS-WHtR	0.97 (0.91–1.04)	0.409	0.92 (0.85–1.00)	0.048	0.98 (0.90–1.06)	0.568
BHGS-WHtR	0.97 (0.91–1.04)	0.371	0.92 (0.85–0.99)	0.034	0.97 (0.90–1.05)	0.479

OR and p values were obtained from the crude and adjusted analyses using complex sample binary logistic regression. Odds ratios were estimated with 95% confidence intervals.

Model 1 was adjusted for age.

Model 2 was adjusted for residential area, marital status, education level, occupation, household income, alcohol consumption, smoking status, muscular exercise, walking exercise, blood pressure, menopause, and age.

*HGS* handgrip strength, *DHGS* dominant hand grip strength, *BHGS* both hand grip strength, *HT* height, *WT* weight, *BMI* body mass index, *WC* waist circumference, *WHtR* waist-to-height ratio, *OR* odds ratio, *CI* confidence interval.

**Table 3:** Demographic characteristics of the subjects in this study.

Variables	Men			Women		
	Nonanemic	Anemic	p value	Nonanemic	Anemic	p value
Subjects (n)	9711	570		12,103	1638	
Age (years)	51.89 ± 0.19	66.56 ± 0.61	< 0.001	52.55 ± 0.19	51.85 ± 0.44	0.103
Residential area**			0.138			0.726
Urban	83.12 (1.15)	80.20 (2.20)		84.21 (1.06)	83.80 (1.50)	
Rural	16.88 (1.15)	19.80 (2.20)		15.79 (1.06)	16.20 (1.50)	
Marital status***			< 0.001			0.048
Married	93.83 (0.29)	85.20 (1.90)		81.44 (0.46)	79.12 (1.19)	
Single (widowed, divorced, etc.)	6.17 (0.29)	14.80 (1.90)		18.56 (0.46)	20.88 (1.19)	
Education level***			< 0.001			< 0.001
< = Elementary school	11.35 (0.40)	30.40 (2.30)		22.24 (0.53)	23.15 (1.28)	
Middle school	10.19 (0.38)	16.60 (1.80)		11.01 (0.35)	7.59 (0.75)	
High school	31.28 (0.63)	33.80 (2.50)		33.40 (0.57)	31.03 (1.36)	
> = University	47.18 (0.84)	19.30 (2.00)		33.35 (0.68)	38.23 (1.55)	
Occupation***			< 0.001			0.003
White-collar worker	20.30 (0.60)	7.16 (1.26)		11.18 (0.36)	12.90 (1.02)	
Office worker	15.80 (0.50)	4.99 (1.09)		8.30 (0.31)	10.18 (0.93)	
Service worker	10.80 (0.40)	7.58 (1.50)		16.04 (0.44)	13.54 (0.98)	
Farmer or fisher	5.00 (0.40)	6.83 (1.27)		2.63 (0.24)	1.32 (0.27)	
Blue-collar worker	25.20 (0.60)	12.07 (1.75)		3.14 (0.20)	3.40 (0.54)	
Elementary occupations	6.80 (0.30)	12.73 (1.72)		10.02 (0.33)	9.39 (0.86)	
Unemployed (housewife, etc.)	16.00 (0.50)	48.65 (2.43)		48.69 (0.60)	49.27 (1.50)	
Household income***			< 0.001			0.016
Low	10.64 (0.39)	35.90 (2.20)		16.77 (0.48)	20.00 (1.20)	
Middle-low	23.71 (0.56)	28.00 (2.20)		24.76 (0.54)	24.50 (1.30)	
Middle-high	30.93 (0.60)	21.40 (2.00)		28.33 (0.53)	28.50 (1.30)	
High	34.72 (0.78)	14.60 (1.80)		30.14 (0.70)	26.90 (1.40)	
Alcohol consumption***			< 0.001			0.292
Never drinker	3.65 (0.21)	10.39 (1.42)		15.10 (0.40)	16.45 (1.11)	
Former drinker 1 year prior	11.20 (0.39)	25.16 (2.06)		19.80 (0.40)	21.12 (1.13)	
< 1 drink per month	10.55 (0.37)	11.74 (1.61)		23.60 (0.50)	23.88 (1.18)	
1 drink per month	8.80 (0.34)	6.79 (1.30)		10.40 (0.30)	10.62 (0.94)	
2 ~ 4 drinks per month	26.46 (0.54)	20.47 (2.01)		19.00 (0.40)	17.99 (1.12)	
2 ~ 3 drinks per week	25.57 (0.53)	14.46 (1.79)		9.70 (0.30)	8.07 (0.76)	
> = 4 drinks per week	13.76 (0.40)	11.00 (1.54)		2.40 (0.20)	1.87 (0.40)	
Smoking status***			< 0.001			0.059
Everyday	31.77 (0.61)	17.20 (1.90)		3.40 (0.20)	2.30 (0.40)	
Sometimes	4.26 (0.26)	2.20 (0.70)		1.50 (0.10)	1.00 (0.30)	
Past	44.13 (0.62)	59.40 (2.40)		5.50 (0.20)	5.50 (0.60)	
Never	19.83 (0.48)	21.20 (2.00)		89.50 (0.30)	91.20 (0.80)	
Muscular exercise***			< 0.001			0.466
Never	68.21 (0.58)	74.90 (2.20)		83.60 (0.40)	85.87 (1.05)	
1 day per week	4.45 (0.24)	2.50 (0.90)		2.60 (0.20)	2.34 (0.43)	
2 days per week	5.89 (0.28)	3.20 (0.90)		3.70 (0.20)	3.46 (0.52)	
3 days per week	6.77 (0.30)	4.20 (1.00)		4.20 (0.20)	3.57 (0.59)	
4 days per week	3.38 (0.22)	0.80 (0.40)		1.50 (0.10)	1.34 (0.35)	
≥ 5 days per week	11.30 (0.38)	14.40 (1.80)		4.40 (0.20)	3.43 (0.54)	
Walking exercise per week (min)*	249.98 ± 4.93	279.18 ± 21.03	0.175	241.08 ± 3.86	215.66 ± 8.17	0.004
Menopause			-			< 0.001
No	-	-		47.10 (0.70)	64.00 (1.50)	
Yes	-	-		52.90 (0.70)	36.00 (1.50)	
Blood pressure						
SBP (mmHg)***	120.78 ± 0.2	123.44 ± 0.86	0.002	116.96 ± 0.22	115.94 ± 0.53	0.066
DBP (mmHg)***	79.01 ± 0.13	70.37 ± 0.56	< 0.001	74.42 ± 0.12	71.64 ± 0.27	< 0.001
Biochemical indices						
Hemoglobin (g/dL)***	15.36 ± 0.01	11.96 ± 0.06	< 0.001	13.41 ± 0.01	10.93 ± 0.03	< 0.001
RBCs (mil/µL)***	4.94 ± 0.01	4.03 ± 0.03	< 0.001	4.40 ± 0.00	4.01 ± 0.01	< 0.001
Anthropometrics						
Height (cm)***	170.77 ± 0.09	166.95 ± 0.30	< 0.001	157.41 ± 0.07	157.27 ± 0.20	0.499
Weight (kg)***	72.00 ± 0.14	64.52 ± 0.49	< 0.001	58.63 ± 0.10	56.83 ± 0.23	< 0.001
Body mass index (kg/m^2^)***	24.64 ± 0.04	23.11 ± 0.16	< 0.001	23.67 ± 0.04	22.98 ± 0.09	< 0.001
Waist circumference (cm)***	87.18 ± 0.10	84.84 ± 0.45	< 0.001	80.08 ± 0.13	78.18 ± 0.26	< 0.001
Waist-to-height ratio**	0.51 ± 0.00	0.51 ± 0.00	0.421	0.51 ± 0.00	0.50 ± 0.00	< 0.001
Dominant hand			0.794			0.468
Right	89.14 (0.40)	88.40 (1.50)		89.90 (0.30)	88.78 (0.95)	
Left	4.86 (0.26)	4.80 (0.90)		4.40 (0.20)	4.71 (0.62)	
Both	6.00 (0.30)	6.80 (1.30)		5.70 (0.20)	6.51 (0.76)	
Absolute HGS						
Dominant HGS (kg)***	38.97 ± 0.11	30.84 ± 0.38	< 0.001	22.64 ± 0.07	21.85 ± 0.16	< 0.001
Both HGS (kg)***	38.18 ± 0.10	30.38 ± 0.37	< 0.001	22.08 ± 0.07	21.30 ± 0.15	< 0.001
Relative HGS						
DHGS-HT (kg/height)***	0.23 ± 0.00	0.18 ± 0.00	< 0.001	0.14 ± 0.00	0.14 ± 0.00	< 0.001
BHGS-HT (kg/height)***	0.22 ± 0.00	0.18 ± 0.00	< 0.001	0.14 ± 0.00	0.13 ± 0.00	< 0.001
DHGS-WT (kg/weight)***	0.55 ± 0.00	0.48 ± 0.01	< 0.001	0.39 ± 0.00	0.39 ± 0.00	0.313
BHGS-WT (kg/weight)***	0.54 ± 0.00	0.48 ± 0.01	< 0.001	0.38 ± 0.00	0.38 ± 0.00	0.274
DHGS-BMI (kg/BMI)***	1.60 ± 0.00	1.35 ± 0.02	< 0.001	0.98 ± 0.00	0.97 ± 0.01	0.424
BHGS-BMI (kg/BMI)***	1.56 ± 0.00	1.33 ± 0.02	< 0.001	0.95 ± 0.00	0.94 ± 0.01	0.388
DHGS-WC (kg/WC)***	0.45 ± 0.00	0.37 ± 0.01	< 0.001	0.29 ± 0.00	0.28 ± 0.00	0.315
BHGS-WC (kg/WC)***	0.44 ± 0.00	0.36 ± 0.00	< 0.001	0.28 ± 0.00	0.28 ± 0.00	0.278
DHGS-WHtR (kg/WHtR)***	77.09 ± 0.24	61.68 ± 0.90	< 0.001	45.37 ± 0.17	45.01 ± 0.43	0.407
BHGS-WHtR (kg/WHtR)***	75.52 ± 0.23	60.73 ± 0.86	< 0.001	44.23 ± 0.17	43.86 ± 0.41	0.369

Continuous data are represented as the mean ± SE (standard error). Categorical data are represented as the percentage (SE).

*SBP* systolic blood pressure, *DBP* diastolic blood pressure, *RBCs* red blood cells, *HGS* hand grip strength, *DHGS* dominant hand grip strength, *BHGS* both hand grip strength, *HT* height, *WT* weight, *BMI* body mass index, *WC* waist circumference, *WHtR* waist-to-height ratio.

*p < 0.05, **p < 0.01, ***p < 0.001. *, **, and *** indicate p values for sex differences between all men and women. P values were obtained from Rao-Scott chi-square tests for categorical variables and from a general linear model for continuous variables between the anemic and nonanemic groups.

Comparing men and women in terms of HGS indices, although absolute HGS indices were highly related to anemia in both sexes in all models, the strengths of the associations between anemia and absolute and relative HGS indices were high in men but moderate in women. All relative HGS values were associated with anemia in men, but in women, only two relative HGS indices, DHGS-HT and BHGS-HT, were related to anemia in all models. Similar to the absolute HGS indices, the associations of the two relative HGS indices were significantly high in men. Comparing absolute and relative HGS indices, the strengths of the associations of anemia with two absolute HGS indices and two relative indices (those showing the highest association with anemia) were similar.

## Discussion

We examined the association of anemia with absolute and relative HGS indices combined with anthropometric variables in a large Korean population. The main findings of this study are as follows: first, both absolute HGS indices and two relative HGS indices were highly associated with anemia in both sexes. Second, the association between relative HGS indices and anemia was not higher than that between absolute HGS and anemia in both sexes, in contrast to other diseases. Therefore, we recommend the use of absolute HGS rather than relative HGS as risk factors for anemia because it’s the former can be more easily and cost-effectively measured.

Recently, several studies have reported an association between anemia/low hemoglobin levels and HGS for identifying the risk of anemia in various ethnic groups or countries^1,3–18,24^. Hirani et al.^1^ examined the association of low hemoglobin concentrations/anemia with sarcopenia, HGS, walking speed, chair stands, and physical disability in Australian men by both cross-sectional and longitudinal studies. They demonstrated that a 1 g/dL increase in hemoglobin was significantly associated with a decreased risk of all outcomes, and HGS had a strongly positive association with hemoglobin levels in all crude and adjusted models in both cross-sectional and longitudinal analyses^1^. Haslam et al.^3^ tested the relationship between anemia and HGS in very elderly subjects with an average age of 100 years in the US and argued that anemia is related to lower HGS and leg strength. Marzban et al.^11^ examined the association between anemia and HGS in Iran and reported that hemoglobin level was significantly associated with mean HGS and relative HGS (HGS combined with BMI) in both men and women. They also showed that the association between mean HGS and anemia was higher than that between the combined HGS/BMI index and anemia. Additionally, Penninx et al.^4^ assessed the association of anemia with disability and HGS in older persons in Italy and found that anemic subjects showed significantly lower HGS than nonanemic subjects, and anemia was related to disability and poor physical performance. Santos et al.^12^ examined the relationship between anemia and absolute HGS and analyzed the receiver operating characteristic (ROC) curve for discriminating anemia in Brazil. They reported that the HGS index was negatively associated with anemia in women but not in men. Furthermore, they obtained a ROC value of 0.71 in anemia/normal classification based on HGS indices and argued that the HGS index could be used to predict anemia in older women. El Shemy et al.^13^ assessed the influence of anemia on the HGS index between the anemic spastic cerebral palsy group and the nonanemic spastic cerebral palsy group in Egyptian children. They found that anemia had an inverse effect on HGS and functional abilities because children with anemia showed a lower HGS than nonanemic children. Penninx et al.^14^ investigated the impact of anemia in older people in terms of HGS and physical performance in the US. They found that HGS in anemic subjects was lower than that in nonanemic subjects and suggested that anemia in older subjects was an independent risk factor for a decrease in physical function. Fukushima et al.^15^ assessed the influence of hemoglobin level on HGS and physical performance in subjects with hematological malignancies in Japan and reported that although physical function did not differ among low, middle, and high hemoglobin groups, the low hemoglobin group was significantly associated with low HGS and reduced muscle strength. Thein et al.^16^ examined the association of anemia with quality of life, functional status, depression, and HGS in older adults in the US and documented that a significant decline in HGS was evident even for hemoglobin levels below 14 g/dL. Yamada et al.^17^ assessed the relationship between HGS and hemoglobin levels in Japanese women and reported that low hemoglobin may cause low HGS, independent of age, any anthropometric index, nutritional components, and inflammation markers such as C-reactive protein. In Korea, Gi et al.^18^ examined the association between the absolute HGS index and anemia and reported that HGS was highly associated with anemia and that the association was higher in men than in women and higher in older age than in middle age. Our findings are consistent with the results of previous studies, indicating that low hemoglobin or anemia was highly associated with low HGS^1,3–18,24^ and that mean HGS and relative HGS indices were associated with anemia, and the strength of the association of the absolute HGS index was better than that of the relative HGS index^11^. Additionally, our finding is linked to the results of previous studies, indicating that the strengths of the associations of mean HGS and/or relative HGS indices were high in men but moderate in women^11,18^, although Marzban et al.^11^ used only one relative HGS index (HGS/BMI), in contrast to our study evaluating 10 relative HGS indices combined with various anthropometric indices. Additionally, age was highly associated with anemia in men but not in women. Therefore, sex and age showed different associations between anemia and HGS indices and may be effect modifiers. Finally, the results of our study conflict with the results of a previous study by Santos et al.^12^, indicating that the HGS index was negatively associated with anemia in women but not in men. Our findings indicated that HGS was inversely associated with anemia in both men and women.

The exact mechanism of the link between anemia/low hemoglobin concentrations and low HGS, including physical function and disability, is unclear^4,15,25^ due to various and complex biological and pathological factors. We can only speculate a possible mechanism linking HGS and anemia. Low HGS is linked to anemia because low hemoglobin levels reduce oxygen delivery to skeletal muscle and thereby reduce muscle strength and physical function^1,4,15,26^. Additionally, fatigue is an important and common sign of low hemoglobin concentration and anemia, and people with fatigue experience reduced HGS and physical function^4,15,18^. Low hemoglobin is linked to dyspnea, which is speculated to be caused by hypoxia due to a decrease in oxygen delivery^15,18^. Strong symptoms of dyspnea or fatigue may influence functional impairment and decrease physical function^15,27^. Generally, men with normal hemoglobin are more likely to perform higher levels of muscular exercise than those with low hemoglobin levels and are more likely to maintain higher HGS^1^. In our study, the association of muscular exercise significantly differed between nonanemic and anemic men, but it was not different in women, while the strength of the association of walking exercise significantly differed between nonanemic and anemic women, but it was not different in men. Another explanation of the mechanism is that anemia is linked to inflammation^4,11,22^. Inflammation in older people is an important etiology of anemia^7,22^. Anemia in elderly individuals is related to high serum levels of inflammatory markers such as interleukin (IL)-6 and C-reactive protein^4,22^. For example, inflammatory cytokines decrease the life span of red blood cells^5,28^. This chronic inflammation may induce a decrease in physical function and muscle strength^4,11,23^.

This study had some limitations. First, this study was cross-sectional in nature and was thus unable to establish a cause‒effect relationship between anemia and HGS. Second, the data used in this study were collected by surveys of the Korean population and may have been subject to respondent recall bias. To mitigate this respondent recall bias in sociodemographic characteristics and the diagnosis of anemia, the health interview survey was performed through a face-to-face interview by well-trained staff and experts according to established guidelines. Third, we could not consider subtypes of anemia in the statistical analysis because anemia subtype data were not collected as part of the KNHANES study design. Finally, the units of the absolute HGS index (kg) and relative HGS indices (kg/BMI, kg/height, kg/WC, and kg/WHtR) are different. Despite these limitations, the statistical results in this study were robust because the very large KNHANES dataset includes a nationally representative sample of the Korean population. To our knowledge, this is the first study to compare absolute HGS and relative HGS indices in terms of the association with anemia in a large population‐based investigation and to report that there is no need to use relative HGS indices for assessing the risk of anemia due to the simple, easy, and cost-effective measurement of the absolute indices.

## Methods

### Study population and data sources

**Figure 1:**
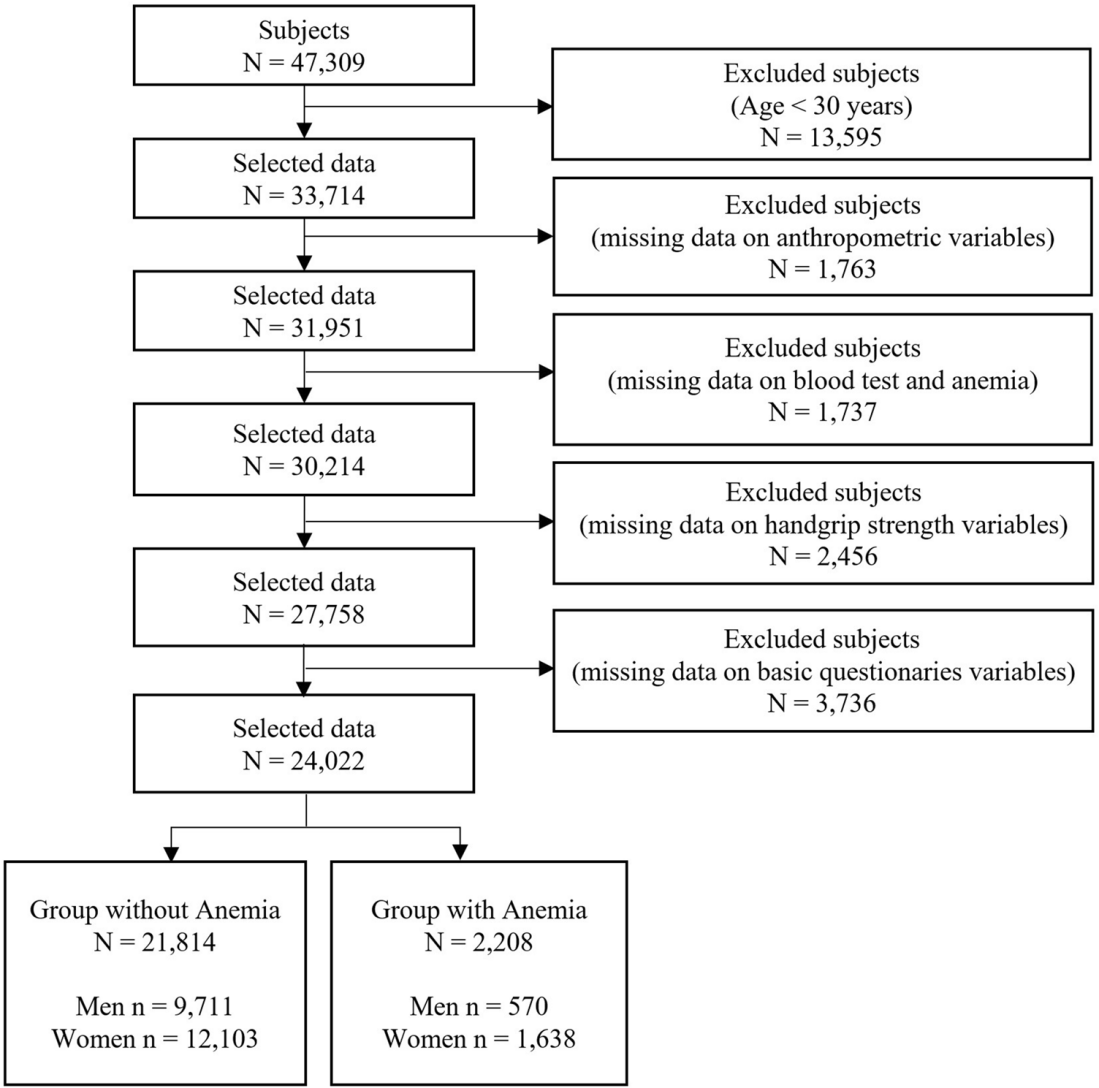
Flowchart of the sample selection procedure used in this study.

In this large-scale cross-sectional study, we utilized the dataset from the Korean National Health and Nutrition Examination Survey (KNHANES), which is conducted annually by the Korea Disease Control and Prevention Agency (KDCA), to investigate the health status, health behavior, and food and nutrient intake of the Korean population. The KNHANES is a nationwide survey conducted to produce statistics that are nationally representative and reliable^29–31^. In this study, we used data from 2014 to 2019, which included a total of 47,309 subjects (men = 21,566, women = 25,743) who completed health surveys and health examinations. All participants in the survey submitted written informed consent. KNHANES was conducted with the approval of the Institutional Review Board of the KDCA (IRB: 2013-07CON-03-4C, 2013-12EXP-03-5C, 2018-01-03-P-A, 2018-01-03-C-A)^31^. In addition, this study based on the KNHANES dataset was approved by the Institutional Review Board of the Korea Institute of Oriental Medicine (IRB No. I-2209/009-001). The study subjects were adults aged 30 years and older, and a total of 24,022 participants (men = 10,281, women = 13,741) with no errors in their analysis variables were finally selected. Figure 1 shows the procedure for selecting the study subjects. This study was conducted in accordance with the Helsinki Declaration, and all analytical methods were performed in accordance with the guidelines and regulations of the Korea Disease Control and Prevention Agency^29–31^.

### Definitions

Anemia is defined based on the hemoglobin levels collected after an 8-h fast. According to the standards of the World Health Organization (WHO)^32^, anemia is defined as a hemoglobin concentration in the blood of less than 13.0 g/dL for men, less than 11.0 g/dL for pregnant women, and less than 12.0 g/dL for nonpregnant women.

### Covariates

All subjects reported the following sociodemographic characteristics. Residential area was dichotomized into rural and urban. Education was classified into four levels: elementary school or less, middle school, high school, and college or above. Marital status was dichotomized into married and single (including widowed, divorced). House income was divided into four levels based on the average monthly income. Alcohol consumption was divided into seven categories based on drinking frequency in the past year. Smoking status was classified into four groups based on the frequency of smoking: “every day”, “sometimes”, “past”, and “never”. Muscular exercise was classified into six categories based on the response to the question "How many days did you perform strength exercises such as push-ups, sit-ups, dumbbells, weightlifting, and pull-ups in the past week?". Walking exercise was expressed as the time walked in minutes per week. Occupation was classified into seven categories. Blood pressure (BP) was presented as a continuous value and was measured as described in the Measurement subsection. Detailed information, including age and all covariates, is described in Table 3. All covariates used in this study were identified in previous studies^1,3,6,11,14,17–20^.

### Measurement

In this study, we analyzed the relationship between anemia and HGS and anthropometrics. HGS was measured using a digital grip strength dynamometer (T.K. K 5401, Japan); subjects were excluded if they had functional limitations or discomfort due to recent hand/wrist surgery or pain within the past 3 months. Measurements were taken with the feet shoulder-width apart, the subject standing facing forward with a straight back and shoulders relaxed, and arms hanging naturally without touching the torso or bending. HGS was measured three times for each hand, alternating hands with a one-minute rest period between each measurement. Absolute HGS was presented as the average value of the dominant hand grip strength (DHGS) measured on the dominant hand and the both hand grip strength (BHGS) measured on both hands. Relative HGS was calculated by dividing absolute HGS by height, weight, BMI, waist circumference (WC), and waist-to-height ratio (WHtR). Anthropometric variables such as height and weight were measured using an automatic measuring device (JENIX DS-102, Dong Sahn Jenix Co., Seoul, Korea) with a precision of 0.1 cm and 0.1 kg, respectively. BMI was calculated by dividing weight (kg) by height squared (m^2^). WC was measured using a tape measure (Seca 200, Hamburg, Germany) with a precision of 0.1 cm, while WHtR was calculated by dividing WC by height. SBP and DBP were measured three times using a standard mercury sphygmomanometer (Baumanometer Wall Unit 33(0850), USA), and the average of the second and third measurements was used. The blood samples used to define anemia were obtained from subjects who had fasted for more than 8 h. Red blood cells and hemoglobin were measured using hydrodynamic focusing DC detection and SLS hemoglobin detection methods with an XL-9000 (Sysmex, Hyogo, Japan)^30,31^.

### Statistical analysis

The KNHANES data were obtained using a complex sample design, and therefore, all analyses were performed using a complex sample analysis (IBM SPSS Statistics version 21, IBM SPSS Inc., Chicago, IL, USA) accounting for weighting, stratification, and clustering variables according to the KDCA guidelines. Statistical significance was set at α = 0.05. The characteristics of male and female subjects with and without anemia are described using percentages and standard errors for categorical variables and means and standard errors for continuous variables. Differences in analyzed variables by sex were evaluated using t tests for continuous variables based on a general linear model and Rao-Scott chi-square tests for categorical variables. The characteristics of the study population are presented in Table 3. We analyzed the association of anemia with anthropometric indices, absolute HGS indices, and relative HGS indices using a binary logistic regression model with odds ratios and 95% confidence intervals after standardizing the data. We created three models based on adjustment variables: the crude model with no adjustments, Model 1 with adjustments for age, and Model 2 with adjustments for age, residential area, marital status, education level, occupation, household income, alcohol consumption, smoking status, muscular exercise, and walking exercise in men and all of the above plus menopause in women.

## Data Availability

Data used in this study are available from the Korea National Health and Nutrition Examination Survey (KNHANES) conducted by the Korea Centers for Disease Control and Prevention (KCDC). Anyone can freely access the data (https://knhanes.kdca.go.kr/knhanes/sub03/sub03_02_05.do).

## References

[1] Hirani V (2016). Low hemoglobin concentrations are associated with sarcopenia, physical performance, and disability in older Australian men in cross-sectional and longitudinal analysis: The concord health and ageing in men project. J. Gerontol. A.

[2] Boogaerts M, Coiffier B, Kainz C (2003). Impact of epoetin beta on quality of life in patients with malignant disease. Br. J. Cancer.

[3] Haslam A (2012). Associations between anemia and physical function in Georgia centenarians. J. Am. Geriatr. Soc..

[4] Penninx BW (2004). Anemia is associated with disability and decreased physical performance and muscle strength in the elderly. J. Am. Geriatr. Soc..

[5] Chaparro CM, Suchdev PS (2019). Anemia epidemiology, pathophysiology, and etiology in low- and middle-income countries. Ann. N. Y. Acad. Sci..

[6] Cesari M (2004). Hemoglobin levels and skeletal muscle: Results from the InCHIANTI study. J. Gerontol. A.

[7] Hess SY (2023). Accelerating action to reduce anemia: Review of causes and risk factors and related data needs. Ann. N. Y. Acad. Sci..

[8] Kassebaum NJ (2014). A systematic analysis of global anemia burden from 1990 to 2010. Blood.

[9] Lee BJ, Kim JY (2016). Identification of hemoglobin levels based on anthropometric indices in elderly Koreans. PLoS ONE.

[10] Balarajan Y, Ramakrishnan U, Ozaltin E, Shankar AH, Subramanian SV (2011). Anaemia in low-income and middle-income countries. Lancet.

[11] Marzban M (2021). Association between anemia, physical performance and cognitive function in Iranian elderly people: Evidence from Bushehr Elderly Health (BEH) program. BMC Geriatr..

[12] Santos PHS, Carmo ÉA, Carneiro JAO, Nery AA, Casotti CA (2019). Handgrip strength: An effective screening instrument for anemia in the elderly women. Public Health Nurs..

[13] El Shemy SA, Amer FE, Madani HA (2019). Impact of iron deficiency anemia on functional abilities and muscle strength in children with spastic cerebral palsy. Pak. J. Biol. Sci..

[14] Penninx BW (2003). Anemia and decline in physical performance among older persons. Am. J. Med..

[15] Fukushima T (2019). Influence of hemoglobin level on muscle and physical functions, activities of daily living, and quality of life in patients with hematological malignancies. Integr. Cancer Ther..

[16] Thein M (2009). Diminished quality of life and physical function in community-dwelling elderly with Anemia. Medicine.

[17] Yamada E (2015). Low haemoglobin levels contribute to low grip strength independent of low-grade inflammation in Japanese elderly women. Asia Pac. J. Clin. Nutr..

[18] Gi YM, Jung B, Kim KW, Cho JH, Ha IH (2020). Low handgrip strength is closely associated with anemia among adults: A cross-sectional study using Korea National Health and Nutrition Examination Survey (KNHANES). PLoS ONE.

[19] Mainous AG, Tanner RJ, Anton SD, Jo A (2015). Grip strength as a marker of hypertension and diabetes in healthy weight adults. Am. J. Prev. Med..

[20] Qaisar R, Karim A, Muhammad T (2020). Circulating biomarkers of handgrip strength and lung function in chronic obstructive pulmonary disease. Int. J. Chron. Obstruct. Pulmon. Dis..

[21] Huang DD (2022). Global leadership initiative in malnutrition (GLIM) criteria using hand-grip strength adequately predicts postoperative complications and long-term survival in patients underwent radical gastrectomy for gastric cancer. Eur. J. Clin. Nutr..

[22] Olivares M, Hertrampf E, Capurro MT, Wegner D (2000). Prevalence of anemia in elderly subjects living at home: Role of micronutrient deficiency and inflammation. Eur. J. Clin. Nutr..

[23] Ferrucci L (2002). Change in muscle strength explains accelerated decline of physical function in older women with high interleukin-6 serum levels. J. Am. Geriatr. Soc..

[24] Öztorun HS (2018). The impact of treatment for iron deficiency and iron deficiency anemia on nutritional status, physical performance, and cognitive function in geriatric patients. Eur. Geriatr. Med..

[25] Joosten E, Detroyer E, Milisen K (2016). Effect of anaemia on hand grip strength, walking speed, functionality and 1 year mortality in older hospitalized patients. BMC Geriatr..

[26] Dodd SL, Powers SK, Brooks E, Crawford MP (1993). Effects of reduced O_2_ delivery with anemia, hypoxia, or ischemia on peak VO_2_ and force in skeletal muscle. J. Appl. Physiol..

[27] Given BA, Given CW, Sikorskii A, Hadar N (2007). Symptom clusters and physical function for patients receiving chemotherapy. Semin. Oncol. Nurs..

[28] Wang CY, Babitt JL (2016). Hepcidin regulation in the anemia of inflammation. Curr. Opin. Hematol..

[29] Korea Disease Control and Prevention Agency. *The Sixth Korea National Health and Nutrition Examination Survey (KNHANES VI-2,3)* (Korea Disease Control and Prevention Agency, 2015).

[30] Korea Disease Control and Prevention Agency. *The Seventh Korea National Health and Nutrition Examination Survey (KNHANES VII-1,2,3)* (Korea Disease Control and Prevention Agency, 2018).

[31] Korea Disease Control and Prevention Agency. *The Eighth Korea National Health and Nutrition Examination Survey (KNHANES VIII-1)* (Korea Disease Control and Prevention Agency, 2019).

[32] World Health Organization. *Haemoglobin Concentrations for the Diagnosis of Anaemia and Assessment of Severity (No. WHO/NMH/NHD/MNM/11.1)* (World Health Organization, 2011).

